# A Novel Concept Acquisition Approach Based on Formal Contexts

**DOI:** 10.1155/2014/136324

**Published:** 2014-07-23

**Authors:** Ting Qian, Ling Wei

**Affiliations:** School of Mathematics, Northwest University, Xi'an, Shaanxi 710069, China

## Abstract

As an important tool for data analysis and knowledge processing, formal concept analysis (FCA) has been applied to many fields. In this paper, we introduce a new method to find all formal concepts based on formal contexts. The amount of intents calculation is reduced by the method. And the corresponding algorithm of our approach is proposed. The main theorems and the corresponding algorithm are examined by examples, respectively. At last, several real-life databases are analyzed to demonstrate the application of the proposed approach. Experimental results show that the proposed approach is simple and effective.

## 1. Introduction

Formal concept analysis (FCA), proposed by Wille in 1982 [1], is a field of applied mathematics based on the mathematization of concept and conceptual hierarchy. It thereby activates mathematical thinking for conceptual data analysis and knowledge processing. FCA starts with a formal context defined as a triple containing an object set, an attribute (property) set, and a binary relation between the object set and the attribute set. A formal concept is a pair (object subset, attribute subset) induced by the binary relation, and a concept lattice is an ordered hierarchical structure of formal concepts. A formal context in FCA corresponds to a special information system with input data being two-valued in rough set theory [2].

Most of the researches on FCA concentrate on the following topics: construction and pruning algorithm of concept lattices [3, 4]; relationship between FCA and rough sets [5–10]; acquisition of rules [11, 12]; reduction of concept lattices [6, 10, 13]. FCA is also proved to be useful in many fields, such as the organization of web search results on a hierarchical structure of concepts based on common topics [14], information retrieval [15, 16], hierarchical analysis of software code [17–19], visualization in software engineering [19, 20], detecting suspects in human traficking [21], analysis of questionnaire data [22], and mining gene expression data [23]. Further references to applications of FCA can be found in [14, 24].

Formal concepts are very important notions of FCA. And intents and extents are also very important elements of formal concepts. The set of intents (extents) is isomorphic to the corresponding concept lattice under the order relationship “⊇” (“⊆”). So, if the set of intents is determined, the corresponding concept lattice is identified. Thus, obtaining all intents or extents is very important. Generally, the basic way to obtain all intents or extents is via their definitions. If there are *n* objects, then we should calculate 2^*n*^ times to obtain all intents. Obviously, the computational costing is very huge. To solve this problem, we give a new method to obtain all intents. And correspondingly, the formal concepts are determined.

This paper is organized as follows. In Section 2, we briefly review some basic notions related to FCA. In Section 3, a novel concept acquisition approach is introduced and some related conclusions are given. In Section 4, the corresponding algorithm is proposed and experimental results are shown to illustrate the validity of our method. Finally, conclusions are drawn in Section 5.

## 2. Preliminaries

In this section, we recall some basic notions and properties in FCA.

**Definition 1 (see [24]).**

A formal context (*G*, *M*, *I*) consists of two sets *G* and *M* and a relation *I* between *G* and *M*. The elements of *G* are called the objects and the elements of *M* are called the attributes of the context. In order to express that an object *g* is in a relation *I* with an attribute *m*, we write *gIm* or (*g*, *m*) ∈ *I* and read it as “the object *g* has the attribute* m*.”

With respect to a formal context (*G*, *M*, *I*), Ganter and Wille [24] defined a pair of dual operators for any *A*⊆*G* and *B*⊆*M* by

(1) $$\begin{array}{c} A^{*}=\left\{m\in M\;|\;gIm\;\forall g\in A\right\},\\ B^{\prime }=\left\{g\in G\;|\;gIm\;\forall m\in B\right\}. \end{array}$$

A formal context is called canonical if  ∀*g* ∈ *G*, g* ≠ *∅*, g* ≠ *M*, and ∀*m* ∈ *M*, *m*′ ≠ *∅*, *m*′ ≠ *G*. We assume that all the formal contexts we study in the sequel are finite and canonical.

Let (*G*, *M*, *I*) be a formal context. ∀*A*_1_, *A*_2_, *A*⊆*G*, ∀*B*_1_, *B*_2_, *B*⊆*M*; the following properties hold.

1. *A*_1_⊆*A*_2_⇒*A*_2_*⊆*A*_1_*,   *B*_1_⊆*B*_2_⇒*B*_2_′⊆*B*_1_′.
2. *A*⊆*A* ^∗′^,  *B*⊆*B* ^′∗^.
3. A* = *A* ^∗′∗^, *B*′ = *B* ^′∗′^.
4. *A*⊆*B*′⇔*B*⊆A*.
5. (*A*_1_∪*A*_2_)* = *A*_1_*∩*A*_2_*, (*B*_1_∪*B*_2_)′ = *B*_1_′∩*B*_2_′.
6. (*A*_1_∩*A*_2_)*⊇*A*_1_* ∪ *A*_2_*, (*B*_1_∩*B*_2_)′⊇*B*_1_′ ∪ *B*_2_′.

If A* = *B* and *B*′ = *A*, then (*A*, *B*) is called a formal concept, where *A* is called the extent of the formal concept and *B* is called the intent of the formal concept. For any *g* ∈ *G*, a pair (*g* ^∗′^, g*) is a formal concept and is called an object concept. Similarly, for any *m* ∈ *M*, a pair (*m*′, *m* ^′∗^) is a formal concept and is called an attribute concept. The family of all formal concepts of (*G*, *M*, *I*) forms a complete lattice that is called the concept lattice and is denoted by *L*(*G*, *M*, *I*). For any (*A*_1_, *B*_1_), (*A*_2_, *B*_2_) ∈ *L*(*G*, *M*, *I*), the partial order is defined by

(2) $$\begin{array}{c} \left(A_{1},B_{1}\right)\leq \left(A_{2},B_{2}\right)⟺A_{1}\subseteq A_{2}\left(⟺B_{1}⊇B_{2}\right). \end{array}$$

And the infimum ∧ and supremum ∨ of (*A*_1_, *B*_1_) and (*A*_2_, *B*_2_) are defined by

(3) $$\begin{array}{c} \left(A_{1},B_{1}\right)\wedge \left(A_{2},B_{2}\right)=\left(A_{1}\cap A_{2},{\left(B_{1}\cup B_{2}\right)}^{\prime *}\right),\\ \left(A_{1},B_{1}\right)\vee \left(A_{2},B_{2}\right)=\left({\left(A_{1}\cup A_{2}\right)}^{*\prime },B_{1}\cap B_{2}\right), \end{array}$$

respectively.

**Definition 2 (see [10]).**

Let (*G*, *M*, *I*) be a formal context. |*G*| < *∞* and |*M*| < *∞*. Denote *L*_*M*_(*G*, *M*, *I*) = {*Y*∣*Y*⊆*M* and *Y* is an intent of (*G*, *M*, *I*)}.

**Example 3 (see [10]).**

**Figure 1:**
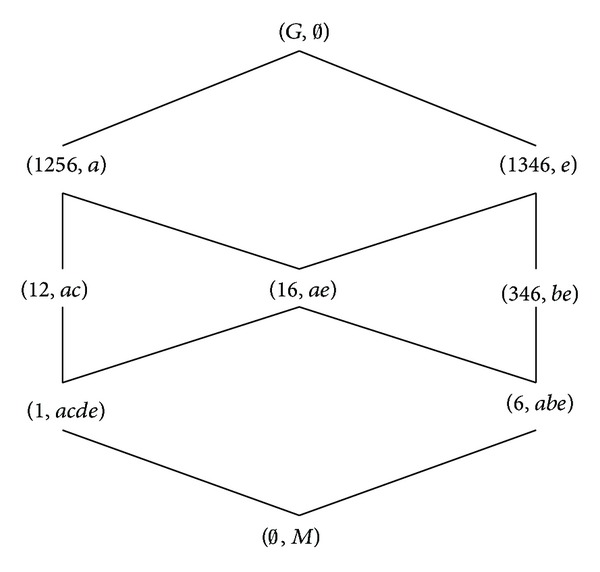
*L*(*G*, *M*, *I*) of Example 3.

Table 1 is a formal context (*G*, *M*, *I*). *G* = {1,2, 3,4, 5,6} is an object set and *M* = {*a*, *b*, *c*, *d*, *e*} is an attribute set. The corresponding concept lattice *L*(*G*, *M*, *I*) is shown in Figure 1, in which every set is denoted directly by listing its elements except *G*, *M*, and *∅*.

## 3. A Novel Concept Acquisition Approach

The basic way to obtain all intents or extents is via their definitions. If there are *n* objects, then we should calculate 2^*n*^ times to get all intents. Obviously, the amount of computation is very large. So our paper presents a new approach to solve the problem. In this section, we give this new method and some theorems to explain its rationality and validity.

Before giving the method, we firstly propose a related definition.

**Definition 4 .**

Let (*G*, *M*, *I*) be a formal context. |*G* | <*∞*, |*M* | <*∞*. Denote *α*_*n*_ = {*X*∣*X*⊆*G* and |*X* | = *n*}, *β*_*n*_ = {*Y*∣*Y*⊆*M*, *Y* = X* and *X* ∈ *α*_*n*_}, *n* = 1,2,…, |*G*|, where |·| presents the cardinal of a set.

Since the method in this paper is aimed at obtaining all intents, we use subsets *α*_*n*_ of *G* to determine subsets *β*_*n*_ of *M*. On the contrary, if we want to obtain all extents, the subsets of *M* can be used to determine subsets of *G*. This point has been illustrated in the sequel.

**Theorem 5 .**

*β*_1_ is an intent of an object concept.

**Proof**

The proof is immediately obtained from Definitions 1 and 4.

**Theorem 6 .**

If there exists *n* (*n* = 1,2,…, |*G*|) such that *β*_*n*+1_⊆*β*_*n*_, then *β*_*n*+2_⊆*β*_*n*+1_.

**Proof**

Suppose *Y* ∈ *β*_*n*+2_. By Definition 4, there exists *X*_*n*+2_ ∈ *α*_*n*+2_ such that *Y* = *X*_*n*+2_*.

Since *X*_*n*+2_ ≠ *∅*, there exists *a* ∈ *X*_*n*+2_ such that *Y* = *X*_*n*+2_* = (*X*_*n*+2_∖{*a*}∪{*a*})* = (*X*_*n*+2_∖{*a*})*∩{*a*}*. Noting that |*X*_*n*+2_∖{*a*}| = *n* + 1, we have *X*_*n*+2_∖{*a*} ∈ *α*_*n*+1_. Moreover, from *β*_*n*+1_⊆*β*_*n*_, we know that there exists *X*_*n*_ ∈ *α*_*n*_ satisfying (*X*_*n*+2_∖{*a*})* = *X*_n_*; that is, *Y* = *X*_n_*∩{*a*}* = (*X*_*n*_∪{*a*})*.

Now, we discuss two cases to prove *Y* ∈ *β*_*n*+1_.

The one case is that *a* ∉ *X*_*n*_. In this case, |*X*_*n*_ ∪ {*a*}| = *n* + 1. Thus, *Y* = *X*_n_*∩{*a*}* = (*X*_*n*_∪{*a*})* ∈ *β*_*n*+1_.

The other one is that *a* ∈ *X*_*n*_. In this case, *Y* = *X*_n_*∩{*a*}* = *X*_n_*. Because *X*_*n*+2_∖*X*_*n*_ ≠ *∅*, there exists *b* ∈ *X*_*n*+2_∖*X*_*n*_ such that *X*_*n*_⊆*X*_*n*_ ∪ {*b*}⊆*X*_*n*_ ∪ *X*_*n*+2_. Therefore, we have *X*_n_*⊇(*X*_*n*_∪{*b*})*⊇(*X*_*n*_∪*X*_*n*+2_)*; that is, *X*_n_*⊇(*X*_*n*_∪{*b*})*⊇*X*_n_*∩*X*_*n*+2_*. That means, *Y*⊇(*X*_*n*_∪{*b*})*⊇*Y*. Therefore, we have *Y* = (*X*_*n*_∪{*b*})*. Thereby, we can obtain *Y* ∈ *β*_*n*+1_.

To sum up the above two cases, *β*_*n*+2_⊆*β*_*n*+1_ holds.

Theorem 6 guarantees the convergence of Algorithm 2 involved in the sequel.

**Corollary 7 .**

If there exists *n* (*n* = 1,2,…, |*G*|) such that *β*_*n*+1_⊆*β*_*n*_, then for any *m*, *m* ≥ *n*, we have *β*_*m*+1_⊆*β*_*m*_⊆*β*_*n*_.

**Proof**

According to the condition *β*_*n*+1_⊆*β*_*n*_, we have *β*_*n*+2_⊆*β*_*n*+1_ by Theorem 6. Using Theorem 6 repeatedly, we can easily obtain the following results: *β*_*m*+1_⊆*β*_*m*_⊆*β*_*m*−1_⊆*β*_*m*−2_⊆⋯⊆*β*_*n*+1_⊆*β*_*n*_.

**Theorem 8 .**

Suppose *Y* ∈ *β*_*k*_, 1 ≤ *k* ≤ |*G*|, 1 < *m* < *k*; if *Y* ∉ *β*_*m*_, then we have *Y* ∉ *β*_*m*−1_.

**Proof**

We will adopt the proof by contradiction.

Suppose *Y* ∈ *β*_*m*−1_; there is *X*_*m*−1_ ∈ *α*_*m*−1_ satisfying *Y* = *X*_*m*−1_*; according to the condition and Definition 4, there is *X*_*k*_ ∈ *α*_*k*_ satisfying *Y* = *X*_k_*.

Because *X*_*k*_∖*X*_*m*−1_ ≠ *∅*, there exists *a* ∈ *X*_*k*_∖*X*_*m*−1_ such that |*X*_*m*−1_ ∪ {*a*}| = *m*; that is, *X*_*m*−1_ ∪ {*a*} ∈ *α*_*m*_. Obviously, *X*_*m*−1_⊆*X*_*m*−1_ ∪ {*a*}⊆*X*_*m*−1_ ∪ *X*_*k*_, and thus, *X*_*m*−1_*⊇(*X*_*m*−1_∪{*a*})*⊇(*X*_*m*−1_∪*X*_*n*_)*. That is, *X*_*m*−1_*⊇(*X*_*m*−1_∪{*a*})*⊇*X*_*m*−1_*∩*X*_k_*. That means *Y*⊇(*X*_*m*−1_∪{*a*})*⊇*Y*. Therefore, we have *Y* = (*X*_*m*−1_∪{*a*})*. From Definition 4, we know that *Y* ∈ *β*_*m*_. It is a contradiction with *Y* ∉ *β*_*m*_.

Therefore, *Y* ∉ *β*_*m*−1_ holds.

**Corollary 9 .**

Suppose *Y* ∈ *β*_*k*_, 1 ≤ *k* ≤ |*G*|, and *Y* ∉ *β*_*k*−1_,*β*_*k*_⊆*β*_*k*−1_; then for any *m*, 1 ≤ *m* < *k*, *Y* ∉ *β*_*m*_.

**Proof**

Because *Y* ∈ *β*_*k*_, and *Y* ∉ *β*_*k*−1_, we have *Y* ∉ *β*_*k*−2_ by Theorem 8. Using Theorem 8 repeatedly, we have *Y* ∉ *β*_*m*_.

**Theorem 10 .**

Suppose (*G*, *M*, *I*) is canonical; then *β*_*n*+1_⊆*β*_*n*_ if and only if  ∪_*i*=1_^*n*^ *β*_*i*_ ∪ {*M*} = *L*_*M*_(*G*, *M*, *I*).

**Proof**

*  *

*Necessity*. Suppose *β*_*n*+1_⊆*β*_*n*_.

For any *Y* ∈ ∪_*i*=1_^*n*^ *β*_*i*_ ∪ {*M*}, if *Y* = {*M*}, then it is evident that *Y* ∈ *L*_*M*_(*G*, *M*, *I*). If *Y* ≠ {*M*}, then there exists *n*_0_ such that *Y* ∈ *β*_*n*_0__. By Definition 4, there exists *X*_*n*_0__ ∈ *α*_*n*_0__ such that *Y* = *X*_*n*_0__*. Obviously, *Y* ∈ *L*_*M*_(*G*, *M*, *I*). Since *Y* is arbitrary and *M* ∈ *L*_*M*_(*G*, *M*, *I*), we have ∪_*i*=1_^*n*^ *β*_*i*_ ∪ {*M*}⊆*L*_*M*_(*G*, *M*, *I*).

For any *Y* ∈ *L*_*M*_(*G*, *M*, *I*), by Definition 2 and properties of the operator ∗, we have *Y* = (Y*)*, Y*⊆*G*. Without loss of the generality, we can suppose |Y* | = *m*. If *m* ≤ *n*, then *Y* ∈ *β*_*m*_⊆∪_*i*=1_^*n*^ *β*_*i*_ by Definition 4. If  *m* > *n*, from above Corollary 7, we have *Y* ∈ *β*_*m*_⊆*β*_*n*_⊆∪_*i*=1_^*n*^ *β*_*i*_. Since *M* ∈ ∪_*i*=1_^*n*^ *β*_*i*_ ∪ {*M*} and *Y* is arbitrary, we obtain ∪_*i*=1_^*n*^ *β*_*i*_ ∪ {*M*}⊇*L*_*M*_(*G*, *M*, *I*).

Therefore, ∪_*i*=1_^*n*^ *β*_*i*_ ∪ {*M*} = *L*_*M*_(*G*, *M*, *I*).

*Sufficiency*. We assume *β*_*n*+1_⊈*β*_*n*_ and prove ∪_*i*=1_^*n*^ *β*_*i*_ ∪ {*M*} ≠ *L*_*M*_(*G*, *M*, *I*).

If *β*_*n*+1_⊈*β*_*n*_, then there is *Y* ∈ *β*_*n*+1_, but *Y* ∉ *β*_*n*_. From Corollary 9, for any *m*, 1 ≤ *m* < *n*, we have *Y* ∉ *β*_*m*_. So, *Y* ∉ ∪_*i*=1_^*n*^ *β*_*i*_. (*G*, *M*, *I*) is canonical and *Y* ∈ *β*_*n*+1_, so *Y* ≠ *M*. Thus, *Y* ∉ ∪_*i*=1_^*n*^ *β*_*i*_ ∪ {*M*}.

On the other hand, since *Y* ∈ *β*_*n*+1_, by Definition 4, there exists *X*_*n*+1_ ∈ *α*_*n*+1_, such that *Y* = *X*_*n*+1_*. By the definition of *L*_*M*_(*G*, *M*, *I*), *Y* ∈ *L*_*M*_(*G*, *M*, *I*).

That means there exists one set *Y* such that *Y* ∉ ∪_*i*=1_^*n*^ *β*_*i*_ ∪ {*M*}, but *Y* ∈ *L*_*M*_(*G*, *M*, *I*). Therefor, ∪_*i*_^*n*^ *β*_*i*_ ∪ {*M*} ≠ *L*_*M*_(*G*, *M*, *I*).

Theorem 10 gives a sufficient and necessary condition and computation method to find *L*_*M*_(*G*, *M*, *I*). Now, the process to calculate all intents is summarized as follows.* Step  1*. Calculate *α*_1_ and *β*_1_ by Definition 4.* Step  2*. Calculate *α*_2_ and *β*_2_ by Definition 4. If *β*_2_⊆*β*_1_, then the set of intents is *β*_1_ ∪ {*M*}. Otherwise, we proceed Step  3.* Step  3*. Calculate *α*_3_ and *β*_3_ by Definition 4. If *β*_3_⊆*β*_2_, then the set of intents is *β*_1_ ∪ *β*_2_ ∪ {*M*}. Otherwise, calculate *β*_*i*_ (1 ≤ *i* ≤ |*G*|) continuously. The computation needs to stop at *β*_*n*+1_ which exactly meets *β*_*n*+1_⊆*β*_*n*_. Meanwhile, the set of intents is ∪_*i*=1_^*n*^ *β*_*i*_ ∪ {*M*}.

The merit of our method is that we do not need to calculate all *β*_*i*_, 1 ≤ *i* ≤ |*G*| and the computation needs only to stop at *β*_*n*+1_ which exactly meets *β*_*n*+1_⊆*β*_*n*_. Now all the intents have been found and there is no extra computing.

In the following, we use an example in the literature [24] to examine the main results about the new method to find all intents of formal concepts.

The formal context in Table 2 is a minor revision of the famous example, a film “Living Beings and Water” [24]. Since we require all the formal contexts in this paper are canonical, we delete the attribute *a* (water) from the original formal context. The objects are living beings mentioned in the film and are denoted by *G* = {1,2, 3,4, 5,6, 7,8}, where 1 is leech, 2 is bream, 3 is frog, 4 is dog, 5 is spike-weed, 6 is reed, 7 is bean, and 8 is maize. And the attributes in *M* = {*b*, *c*, *d*, *e*, *f*, *g*, *h*, *i*} are the properties which the film emphasizes: *b*: lives in water, *c*: lives on land, *d*: needs chlorophyll to produce food, *e*: two seed leaves, *f*: one seed leaf, *g*: can move around, *h*: has limbs, and *i*: suckles its offspring.

**Figure 2:**
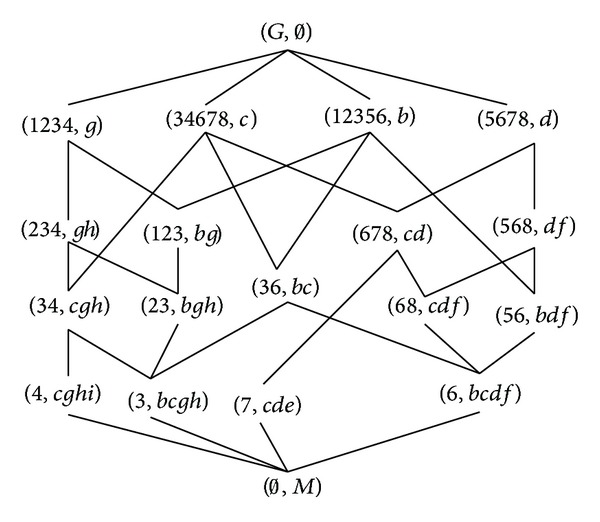
*L*(*G*, *M*, *I*).

The corresponding concept lattice *L*(*G*, *M*, *I*) of this formal context is shown in Figure 2.

We calculate *α*_*i*_ and *β*_*i*_ (*i* = 1,2, 3,…, 8) firstly:

(4) α1={{1},{2},{3},{4},{5},{6},{7},{8}},β1={{b,g},{b,g,h},{b,g,c,h},{c,g,h,i},{b,d,f},   {b,c,d,f},{c,d,e},{c,d,f}},α2={{1,2},{1,3},{1,4},{1,5},{1,6},{1,7},{1,8},{2,3},   {2,4},{2,5},{2,6},{2,7},{2,8},{3,4},{3,5},{3,6},   {3,7},{3,8},{4,5},{4,6},{4,7},{4,8},{5,6},{5,7},   {5,8},{6,7},{6,8},{7,8}},β2={{b,g},{g},{b},∅,{b,g,h},{g,h},{c,g,h},{b,c},{c},   {b,d,f},{d},{d,f},{c,d},{c,d,f}},α3={{1,2,3},{1,2,4},{1,2,5},{1,2,6},{1,2,7},{1,2,8},   {2,3,4},{2,3,5},{2,3,6},{2,3,7},{2,3,8},{3,4,5},   {3,4,6},{3,4,7},{3,4,8},{4,5,6},{4,5,7},{4,5,8},   {5,6,7},{5,6,8},{6,7,8},{1,3,4},{1,3,5},{1,3,6},   {1,3,7},{1,3,8},{2,4,5},{2,4,6},{2,4,7},{2,4,8},   {3,5,6},{3,5,7},{3,5,8},{4,6,7},{4,6,8},{5,7,8},   {1,4,5},{1,4,6},{1,4,7},{1,4,8},{2,5,6},{2,5,7},   {2,5,8},{3,6,7},{3,6,8},{4,7,8},{1,5,6},{1,5,7},   {1,5,8},{2,6,7},{2,6,8},{3,7,8},{1,6,7},   {1,6,8},{2,7,8},{1,7,8}},β3={{b,g},{g},{b},∅,{b,g,h},{g,h},{c,g,h},   {b,c},{c},{b,d,f},{d},{d,f},{c,d},{c,d,f}}.

Similarly, we can calculate *β*_4_, *β*_5_,…, *β*_8_ and find *β*_2_⊇*β*_3_⊇*β*_4_⊇*β*_5_⊇⋯⊇*β*_8_. And we can also know *β*_2_⊈*β*_1_. In fact, we only need to calculate *β*_1_, *β*_2_, *β*_3_. Once we have *β*_3_⊆*β*_2_, but *β*_2_⊈*β*_1_, the computation can be stopped.

According to Theorem 10, the set of all the intents is *β*_1_ ∪ *β*_2_ ∪ {*M*}; that is, *L*_*M*_(*G*, *M*, *I*) = {{*b*, *g*}, {*g*}, {*b*}, *∅*, {*b*, *g*, *h*}, {*g*, *h*}, {*c*, *g*, *h*}, {*b*, *c*}, {*c*}, {*b*, *d*, *f*}, {*d*}, {*d*, *f*}, {*c*, *d*}, {*c*, *d*, *f*}, {*b*, *g*, *c*, *h*}, {*c*, *g*, *h*, *i*}, {*b*, *c*, *d*, *f*}, {*c*, *d*, *e*}, *M*}. These results are easily examined from Figure 2.

## 4. Algorithms and Experiments

### 4.1. Algorithms

Algorithm 1 is given based on Definition 1 completely.

Algorithm 2 is based on our approach presented by Theorem 10. Comparing with Algorithm 1, we add a condition to terminate the program.

The time complexity of Algorithm 2 is analyzed as follows.

Denote *N* = min⁡{|*G* | , |*M*|}; by Definition 4, we know the time complexity of Step I in Algorithms 1 or 2 is *O*(*C*_*N*_^*i*^). So we can get two matters as follows.

1. The time complexity of algorithm is *O*(2^*N*^).
2. Suppose that Algorithm 2 will be terminated in the *k*th step; then the time complexity of Algorithm 2 is *O*(∑_*i*=1_^*i*=*k*^(*C*_*N*_^*i*^)) by Theorem 10. We can easily get *O*(∑_*i*=1_^*i*=*k*^(*C*_*N*_^*i*^)) ≤ *O*(2^*N*^).

We present an example demonstrating performance of Algorithm 2. The database “patient and Ill symptoms” showed in Table 3 comes from UCI Machine Learning Repository [25]. Suppose there are 12 patients which are denoted by 1,…, 12 and 8 symptoms of patients which are denoted by *a*,…, *h*, where *a* is headache, *b* is fever, *c* stands for painful limbs, *d* represents swollen glands in neck, *e* is cold, *f* is stiff neck, *g* is rash, and *h* is vomiting. Input the formal context and run the program; we obtain the set of all intents when *n* = 2:

 {*∅*, {*b*}, {*a*, *b*}, {*b*, *e*}, {*g*}, {*b*, *e*, *g*}, {*a*, *b*, *f*, *h*}, {*a*, *b*, *c*, *e*}, {*a*, *b*, *c*, *d*, *e*, *f*, *g*, *h*}} .

### 4.2. Experimental Results

In this section, we conduct some experiments to compare Algorithm 2 with Algorithm 1. In the experiments, four real life databases we selected are as follows:

1. Living beings and water [24] introduced in Section 4.1.
2. Patients and ill symptoms [25] introduced in Section 4.1.
3. *Bacterial Taxonomy* [26]. Data are presented for 6 species most of whom having data for more than one strain and 16 phenotypic characters (0 and 1). The species are* Escherichia coli* (*ecoli*),* Salmonella typhi* (*styphi*),* Klebsiella pneumoniae* (*kpneu*),* Proteus vulgaris* (*pvul*),* Proteus morganii* (*pmor*), and* Serratia marcesens* (*smar*). The phenotypic characters are H2S, MAN, LYS, IND, ORN, CIT, URE, ONP, VPT, INO, LIP, PHE, MAL, ADO, ARA, and RHA.
4. *Membership of Developing Countries in Supranational Group* [24]. In this data, 130 developing countries are objects. Six properties (group of 77, nonaligned, least developed countries, most seriously affected countries, Organization of Petrol Exporting Countries, and African Caribbean and Pacfic Countries) are attributes.

**Figure 3:**
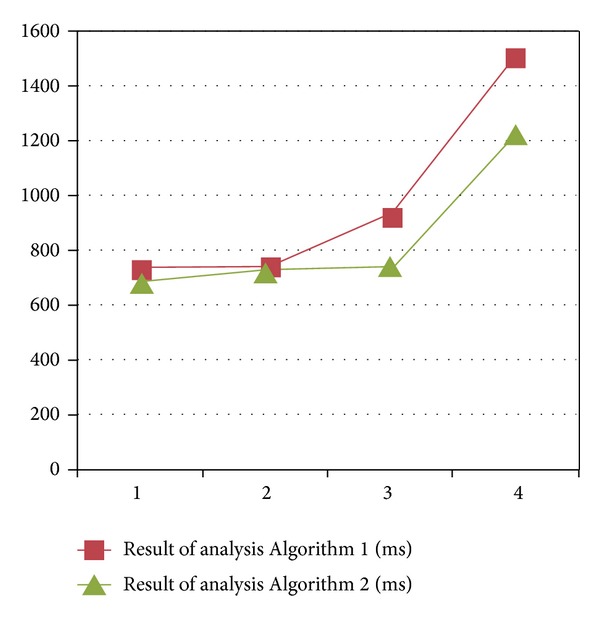
Database analysis.

The results are shown in Table 4 and Figure 3, where Time 1 and Time 2 are the running time of Algorithms 1 and 2, respectively. |*I*| presents the number of intents and the efficiency is equivalent to (Time 1 − Time 2)/Time 1. It can be seen that Algorithm 2 is much more efficient than Algorithm 1 along with the increase of |*I*|.

## 5. Conclusion

To find new methods to solve the difficult problems of the concept lattice construction is a hot problem. Constructing concept lattices is a novel research branch for data processing and data analysis. Different methods play essential roles in different problems. This paper first defines some basic notions. Based on the basic notion of intents, we obtain a new judgment method of finding all intents of formal concepts. Moreover, an example is given to explain the feasibility of this method. At last, we give the corresponding algorithm of this method and do the experiments to illustrate the effectiveness of this method.

For Algorithm 2, we have the following discussion which can be applied to real application. We can compare |*G*| with |*M*| of a formal context. If |*G* | ≤|*M*|, then we use subsets of *G* to determine subsets of *M* and output the set of intents. Otherwise, according to the duality principle, the subsets of *M* can be used to determine subsets of *G* and output the set of extents. We will improve the corresponding algorithm of this method in the future.

## Figures and Tables

**Algorithm 1:**
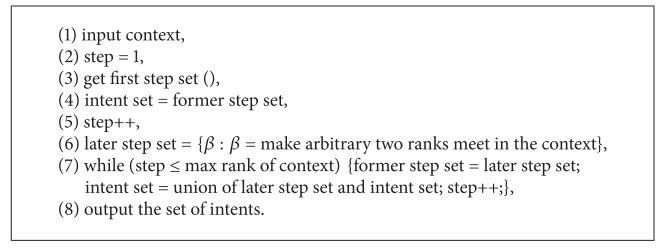


**Algorithm 2:**
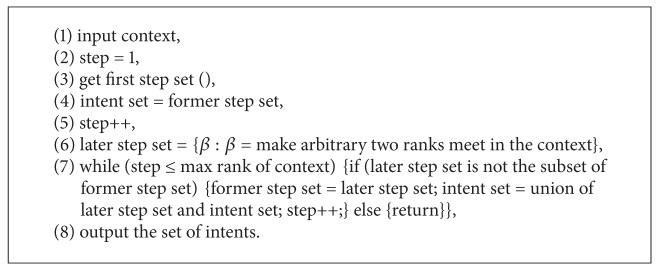


**Table 1:** A formal context (*G*, *M*, *I*).

*G*	*a*	*b*	*c*	*d*	*e*
1	*✗*		*✗*	*✗*	*✗*
2	*✗*		*✗*		
3		*✗*			*✗*
4		*✗*			*✗*
5	*✗*				
6	*✗*	*✗*			*✗*

**Table 2:** Living beings and water (*G*, *M*, *I*).

*G*	*b*	*c*	*d*	*e*	*f*	*g*	*h*	*i*
1	*✗*					*✗*		
2	*✗*					*✗*	*✗*	
3	*✗*	*✗*				*✗*	*✗*	
4		*✗*				*✗*	*✗*	*✗*
5	*✗*		*✗*		*✗*			
6	*✗*	*✗*	*✗*		*✗*			
7		*✗*	*✗*	*✗*				
8		*✗*	*✗*		*✗*			

**Table 3:** Patients and ill symptoms (*G*, *M*, *I*).

*G*	*a*	*b*	*c*	*d*	*e*	*f*	*g*	*h*
1	*✗*	*✗*	*✗*		*✗*			
2	*✗*	*✗*				*✗*		*✗*
3		*✗*			*✗*		*✗*	
4	*✗*	*✗*				*✗*		*✗*
5	*✗*	*✗*	*✗*		*✗*			
6		*✗*			*✗*		*✗*	
7		*✗*			*✗*		*✗*	
8							*✗*	
9	*✗*	*✗*	*✗*		*✗*			
10							*✗*	
11	*✗*	*✗*	*✗*		*✗*			
12	*✗*	*✗*				*✗*		*✗*

**Table 4:** A contrast between algorithms.

Data	|*G*|	|*M*|	|*I*|	Time 1	Time 2	Efficiency
1	8	8	19	733	686	6.4%
2	12	8	9	733	718	2.1%
3	17	16	53	921	733	20.4%
4	130	6	23	1504	1217	19.1%
